# Ketamine Infusion Protocol for the Treatment of Complex Regional Pain Syndrome

**DOI:** 10.7759/cureus.116450

**Published:** 2026-09-17

**Authors:** Evan B Hershman, Tony L Wang

**Affiliations:** 1 Pain Management, Community Memorial Hospital, Ventura, USA

**Keywords:** algodystrophy, causalgia, chronic regional pain syndrome (crps), ketamine, ketamine infusion dosage, ketamine infusion rate, reflex sympathetic dystrophy syndrome (rsds), shoulder-hand syndrome, sudeck's atrophy

## Abstract

This case report presents a 57-year-old gentleman with treatment-resistant complex regional pain syndrome (CRPS) following upper limb amputation. Despite numerous interventions, including medications, nerve blocks, and spinal cord stimulators, his pain remained refractory. Four weekly ketamine infusions were administered, resulting in a notable 50% reduction in CRPS-associated pain persisting for up to four weeks post-treatment. The case demonstrates ketamine infusion therapy's potential in managing refractory CRPS, suggesting it is a promising option for patients unresponsive to conventional treatments. This case particularly highlights the use of ketamine infusion in a non-optimal circumstance with repeated weekly treatments, while most prior research has taken place primarily in an inpatient setting with continuous or daily treatments. Further research is needed to optimize dosing protocols and evaluate long-term efficacy and safety.

## Introduction

Complex regional pain syndrome (CRPS) is a challenging neuropathic disorder characterized by severe and often debilitating chronic pain, following trauma or injury. It predominantly affects limbs and involves sensory, motor, and autonomic dysfunctions, with symptoms including burning pain, swelling, skin changes, and impaired movement. CRPS is typically diagnosed clinically using the Budapest criteria, which were developed to improve diagnostic accuracy over earlier IASP criteria. CRPS presents a significant therapeutic hurdle, as many patients fail to achieve adequate relief from conventional treatments, including physical therapy, nerve blocks, and pharmacological agents; for patients whose pain is refractory to these standard interventions, alternative therapeutic strategies are warranted. Ketamine, originally developed as an anesthetic, has garnered significant attention for its potential in managing various neuropsychiatric and pain disorders, including CRPS [1, 2]. Its mechanism of action involves antagonizing the N-methyl-D-aspartate (NMDA) receptor and enhancing glutamatergic signaling, a unique pharmacological profile that disrupts the central sensitization processes believed to underpin CRPS [3]. By modulating pain perception and potentially resetting maladaptive pain pathways, ketamine has emerged as a promising option in the realm of chronic pain management.

Most of the research investigating ketamine's efficacy for CRPS has involved continuous or repeated daily infusions, often administered in an inpatient setting [4-6]. While these protocols have shown success, they are not always practical for every patient due to constraints like work schedules, cost, and attending physician availability. This limitation highlights the need to explore more flexible, pragmatic dosing schedules. This case highlights the use of repeated outpatient ketamine infusions in refractory CRPS within a literature marked by substantial heterogeneity in dosing, duration, and treatment setting, including both outpatient intermittent protocols and inpatient continuous infusions. Further research is needed to optimize dosing protocols and evaluate long-term efficacy and safety.

## Case presentation

The patient, a 57-year-old gentleman, presented with a complex medical history (Table 1). In 2013, the patient had multiple right-hand carpal tunnel surgeries. Due to complications, he had to have a right upper extremity amputation proximal to the wrist. Consequently, he experienced phantom limb pain in the amputated limb and later developed CRPS in the right upper extremity, which then spread to his entire upper body and eventually to his left lower extremity. In 2020, he had a stroke that caused left hemiparesis; he later recovered and learned how to walk again. In 2021, he had a spinal cord stimulator placed, which was beneficial for his pain, but then was complicated by infection and had to be removed. The patient reported that the pain associated with CRPS was constant, rated 7/10 with a sharp, shooting quality. The pain was aggravated by any activity, standing, walking, lying on his back, or muscle movement and alleviated with ice and heat. The patient had previously undergone numerous interventions to manage his pain, including acupuncture, physical therapy, as well as weekly or bi-weekly stellate ganglion nerve blocks, totaling 55 injections, which provided moderate pain relief for one to two weeks. Medications include gabapentin, oxycodone, tizanidine, baclofen, methocarbamol, cymbalta, and meloxicam.

**Table 1:** Patient Timeline and Intervention Summary

Year	Event/Intervention	Key Details	Outcome
2013	Right-hand carpal tunnel surgeries	Multiple surgeries; resulted in complications.	Right upper extremity amputation, development of phantom limb pain, and later CRPS.
2020	Stroke	_	Caused left hemiparesis; patient recovered and regained walking ability.
2021	Spinal cord stimulator (SCS) placement	_	Initial benefit for pain, but removed due to infection.
2020/2021	Stellate ganglion blocks	55 injections, occurring weekly/bi-weekly.	Moderate pain relief for 1-2 weeks.
Ongoing	Medications	Gabapentin, oxycodone, tizanidine, baclofen, methocarbamol, cymbalta, meloxicam.	Pain constant, rated 7/10, sharp/shooting quality.
2022	Weekly ketamine infusions (4 sessions)	Stepwise infusion protocol over 4 hours, once weekly	50% reduction in CRPS pain after the second infusion. Pain relief persisted for up to 4 weeks post-final infusion.

Intervention and outcome

The patient had previously been given ketamine infusions in the emergency department and various other medical centers for his pain, and it was determined that continuing these infusions would be the most beneficial course of action going forward. The protocol was discussed with the hospital pharmacist and cross-checked with hospital protocol, which states that the maximum dose of ketamine allowed for CRPS indication is 0.5 mg/kg/hr.

The protocol was carried out for a total of four weekly sessions. After applying standard ASA monitors (continuous pulse oximeter, 5-lead ECG, non-invasive BP cuff), 2 mg of midazolam was given intravenously (IV) before the start of infusion by nursing staff. Five minutes after the administration of IV midazolam, IV ketamine infusion was started at a rate of 0.2 mg/kg/hr. After one hour, the infusion rate increased to 0.3 mg/kg/hr for another hour. After two hours of infusion, an additional 2 mg of IV midazolam as well as 4 mg of IV ondansetron were given to the patient for patient-reported anxiety and nausea, respectively. Additionally, the IV ketamine infusion rate was increased to 0.4 mg/kg/hr for one hour. After three hours of infusion, the IV ketamine infusion rate was increased to 0.5 mg/kg/hr for another hour. The infusion was stopped after a total of four hours. 

Following the initial infusion, the patient did not experience much pain relief. However, after his second infusion (starting and continuing with the highest tolerable dose as above), the patient experienced a 50% reduction in pain associated with his CRPS. This benefit persisted through four weekly treatments and lasted for about four weeks after the final infusion, after which pain returned to the pre-treatment baseline of 7/10 (Table 1). No additional ketamine course was given at that time due to patient preference, and he was subsequently lost to follow-up.

## Discussion

Ketamine infusion has long been used in CRPS management, particularly in patients with refractory disease. However, there remains no universally accepted standard protocol for dosing, duration, frequency, treatment setting, or side-effect monitoring, and published regimens vary substantially across outpatient and inpatient practice. While many institution-specific protocols have been established, these have been formulated using a variety of factors not necessarily fully rooted in optimal CRPS pain relief. For example, factors such as cost, available personnel, patient follow-up, and other considerations play a significant role in establishing institution-specific protocols. While these factors are important to think about at a practical level, it is also important to establish an optimal protocol based on CRPS-related pain reduction efficacy. The following studies have attempted to contribute to this determination, but continued research is still needed.

In a double-blind placebo-controlled study, subjects were infused intravenously with normal saline with or without ketamine for four hours (25 ml/h) daily for 10 days. 0.35 mg/kg/h was set as the maximum ketamine infusion rate, with an upper bound of 25 mg/h over a four-hour period. These treatments resulted in clinically significant pain reductions [1].

In a systematic review detailing and analyzing trials in which the effects of ketamine infusion treatment were studied, 14 studies met the inclusion criteria. In these studies, the dosage of ketamine infusion used ranged from 0.15 mg/kg to 7 mg/kg per session, with the primary indication being the treatment of CRPS. In 13 of the studies, ketamine infusion led to a decrease in pain scores and relief of symptoms. However, these trials completed constant infusions or repeated daily infusions for four hours to 10 days in a row [2]. 

A prospective study without placebo control suggested that four days of treatment with ketamine are sufficient for the treatment of CRPS-associated hyperalgesia of the lower extremities. However, for the upper extremities, greater than four days may be necessary. Their treatment protocol included four hours of ketamine infusion per day with a stepwise increase of doses from 60 mg/hr at the beginning of treatment on the first day, progressing to 200 mg/hr by the end of treatment on the fourth day [3].

Other studies detailed long-term (days to weeks) ketamine treatment at an anesthetic level (involving patients anesthetized and ventilated in an intensive care unit), known as a “ketamine coma.” In these studies, treatment was associated with more effective pain relief for longer periods (six or more months) compared to patients undergoing daily or weekly repeated infusions, as with our patient; these articles claim to have effectively intermittently “cured” CRPS [4,5]. However, studies involving these ketamine comas have only come in the form of case reports and open-label, uncontrolled trials, and more in-depth research needs to be completed. Furthermore, these types of treatments carry risk-benefit profile concerns [6].

Additionally, it should be noted that with all ketamine-infusion protocols, there exists concern for hepatotoxicity, and caution should be taken by monitoring liver function tests during infusions, especially if infusions take place over an extended period [7].

The studies outlined above have presented treatment protocols that are highly effective in reducing CRPS-related pain, with the majority recommending daily subanesthetic ketamine infusions spanning three to 10 days. However, this regimen is not practical for every patient. In the case of our patient, his work schedule precluded him from taking consecutive days off for infusions, leading him to opt for weekly sessions instead. Complicating matters further, the attending physician's responsibilities across multiple sites rendered daily oversight unfeasible.

Despite these suboptimal circumstances, it's crucial to highlight that the patient still experienced significant relief from CRPS pain through his weekly ketamine infusions. Although his 50% reduction in pain over four weeks was less than the sustained efficacy observed in some of the aforementioned trials, it's noteworthy that real-life constraints may impact future CRPS patients, hindering their ability to adhere to daily ketamine infusion schedules. Our patient's case underscores the importance of developing and establishing the efficacy of alternative ketamine infusion treatment plans that are practical and effective, even if they are not optimal.

## Conclusions

Ketamine infusion therapy shows promise as a treatment option for individuals suffering from CRPS, particularly those with refractory symptoms unresponsive to conventional therapies. In this case, the patient experienced a notable 50% reduction in CRPS-associated pain following a series of four weekly ketamine infusions, with the relief persisting for up to four weeks after the final treatment. This outcome highlights ketamine's potential to provide substantial and relatively rapid pain relief, even when pragmatic, non-optimal treatment approaches, such as weekly outpatient sessions, are necessary due to real-world constraints. The demonstrated efficacy of a weekly protocol suggests that alternative, flexible infusion schedules should be further developed and investigated for patients unable to adhere to more intensive, daily regimens.

The optimal dosing regimen and duration of ketamine treatment for CRPS remain areas of ongoing investigation. Prior research has shown success with widely varying protocols, from continuous or repeated daily infusions to high-risk, anesthetic-level treatments like the "ketamine coma" approach. Further research is warranted to better understand the efficacy and safety of these modalities. Specifically, controlled trials with larger sample sizes and longer follow-up periods are needed. Comparative studies evaluating different dosing regimens, including the weekly approach presented here, are essential to elucidate the most effective and sustainable strategy for alleviating CRPS-associated pain while minimizing potential risks.
